# *CYP2D6* genetic polymorphisms in Saudi systemic lupus erythematosus patients: A cross-sectional study

**DOI:** 10.15537/smj.2023.44.3.20220581

**Published:** 2023-03

**Authors:** Lena M. Hassen, Maha H. Daghestani, Mohammed A. Omair, Arwa K. Althomali, Fatimah B. Almukaynizi, Ibrahim A. Almaghlouth

**Affiliations:** *From the Department of Zoology (Hassen, Daghestani), College of Sciences; from the Department of Medicine (Hassen, Omair, Almaghlouth), Rheumatology Unit; from the College of Medicine Research Center (Almaghlouth), College of Medicine; and from Prince Naif for Health Research Center (Althomali, Almukaynizi), King Saud University, Riyadh, Kingdom of Saudi Arabia.*

**Keywords:** cytochrome P450, genotyping, risk allele, lupus, clinical features

## Abstract

**Objectives:**

To determine the prevalence of selected single nucleotide polymorphisms (rs1080985, rs28624811, rs1065852, rs28371725, and rs1135840) in *cytochrome* *P450 2D6* (*CYP2D6*) gene among Saudi systemic lupus erythematosus (SLE) patients and to investigate the association between the genetic variants and clinical features of SLE.

**Methods:**

This cross-sectional study was carried out on adult Saudi patients at King Khalid University Hospital, Riyadh, Saudi Arabia. Patients with confirmed SLE based on the 2012 Systemic Lupus International Collaborating Clinics classification criteria were included in the study. Peripheral blood was collected for genomic deoxyribonucleic acid extraction and TaqMan^®^ technologies were used for target genotyping. For statistical analysis, differences in genotype frequencies were determined using the Chi-square test, and the association between the variant genotypes and SLE features was evaluated using logistical regression models.

**Results:**

There were 107 participants included in this study. Overall, the most predominant (23.4%) recessive genotype was AA in rs28624811, and the least prevalent (1.9%) recessive genotype was TT in rs28371725. Moreover, the variant rs1080985 genotypes (GC or CC) were significantly associated with the presence of serositis manifestation (OR=3.15, *p*=0.03), even after adjusting for age and gender. However, the dominant rs28624811 genotype (GG) was associated with renal involvement (OR=2.56, *p*=0.03).

**Conclusion:**

Systemic lupus erythematosus patients carrying *CYP2D6* variants might be considered at risk for certain manifestations of SLE. Further studies are needed to investigate the implication of these genetic variations in clinical outcomes and drug response.

**T**he *cytochrome P450 2D6* (*CYP2D6*) gene region is extensively polymorphic. There are almost 150 variant alleles, predominantly represented by single nucleotide polymorphism (SNP), identified today and cataloged in the database of the Human Cytochrome P450 Allele Nomenclature (now Pharmacogene Variation Consortium). These genetic variations cause phenotypic variations in endogenous and exogenous metabolism leading to large inter-individual variability in metabolic outcomes and drug response.^1^

The *CYP2D6* polymorphism also exhibits substantial inter-ethnic differences. The worldwide data on *CYP2D6* genetic/allelic frequencies revealed that the most frequently observed variant alleles, besides *CYP2D6* ^*^ *1* (a wild-type allele), were *CYP2D6* ^*^ *2*, ^*^ *4*, ^*^ *10*, ^*^ *17*, and ^*^ *41*. Some of the highest reported *CYP2D6* variants are reported among Asian populations, where the most prevalent allele is *CYP2D6* ^*^ *10* (decreased-functional allele) in East Asians. In fact, the present trends for *CYP2D6* ^*^ *10* frequencies pivot between 45-50%, as confirmed by several meta-analysis studies.^2,3^

Despite decades of research, the role of CYP in systemic lupus erythematosus (SLE) is still unknown. Some of the earliest postulated hypotheses include the possibility that an unknown substrate metabolized by CYP may trigger autoimmunogens leading to the generation of immune disorders. It is also possible that alternative CYP metabolism of xenobiotics may contribute to multiple chemical sensitivity syndrome, such as drug-induced lupus. The possibility even extends to which perturbed oxidative reactions due to variant CYP involved in metabolizing endogenous substrate, including arachidonic acids associated with oxidative stress, may play a role in the generation of atherosclerosis and cancer, such that seen in some cases of SLE.^4^

Based on these assumptions, SLE represents an important context under which *CYP2D6* variants may confer heightened risk. However, evidence from the literature shows conflicting results. For example, some studies have reported that the *CYP2D6* ^*^ *4* (odds ratio [OR]=2.0, 95% confidence interval [CI]=[1.17-3.44]; *p*=0.01) and poor metabolizer phenotype (OR=1.78, 95% CI=[1.25-2.53], *p*=0.001) are associated with SLE development.^5^ Other studies, on the other hand, suggested no correlation between *CYP2D6* ^*^ *4* gene mutations and SLE.^6-8^ We believe that these differences are contingent on ethnic differences.

The evidence of ethnicity is an important factor in pharmacogenetics studies. Additionally, the knowledge of *CYP2D6* polymorphism in SLE may help establish a recommendation for considering genetic markers in optimizing care and therapy for lupus patients, particularly in the case of specific organ involvement. Thus, we carried out a study investigating the prevalence of the selected *CYP2D6* SNPs (Table 1) in Saudi SLE patients and evaluating the possible correlation between the variant *CYP2D6* genotypes with the clinical features of SLE.

**Table 1:** - The selected *cytochrome P450 2D6* single nucleotide polymorphisms for this study.

SNP ID/name	Position reference	SNP region	Genetic variation	Functional consequence	Phenotype prediction^*^	Global MAF†
rs1080985 (CYP2D6^*^2)	NC_000022.11:g.42132375G>C	Promotor	2KB upstream variant	No function	-	Samples = 14,996 G = 0.87 (Ref) C = 0.13 (Alt)
rs28624811 (CYP2D6^*^36)	NC_000022.11:g.42131531G>A	Promotor	2KB upstream variant	No function	-	Samples = 55,678 G = 0.66 (Ref) A = 0.34 (Alt)
rs1065852 (CYP2D6^*^4)	NC_000022.11:g.42130692G>A	Exon 1	Missense variant; splice defect	Decreased function	PM	Samples = 48,628 G = 0.79 (Ref) A = 0.21 (Alt)
rs28371725 (CYP2D6^*^41)	NC_000022.11:g.42127803C>T	Intron 6	Intron variant; splice defect	Decreased function	IM-PM	Samples = 43,504 C = 0.91 (Ref) T = 0.09 (Alt)
rs1135840 (CYP2D6^*^10)	NC_000022.11:g.42126611C>G	Exon 9	Missense variant	Decreased function	IM-PM	Samples = 24,724 C = 0.43 (Ref) G = 0.57 (Alt)

* Phenotype prediction is based on Clinical Pharmacogenetics Implementation Consortium recommendations. †Global minor allele frequency is based on Allele Frequency Aggregator project database. Alt: alternative allele, *CYP2D6: cytochrome P450 2D6*, ID: identification, IM: intermediate metabolizers, SNP: single nucleotide polymorphism, MAF: minor allele frequency, PM: poor metabolizers, Ref: reference allele

## Methods

This study was a cross-sectional observational investigation. The study participants were recruited from the National Lupus Prospective Cohort at King Khalid University Hospital (KKUH), Riyadh, Saudi Arabia, between March 2020 and March 2021.

Adult (18 years and older) Saudi patients who were diagnosed with SLE at least one year prior to study enrollment and who had met the 2012 Systemic Lupus International Collaborating Clinics classification criteria were included in our study.^9^

The sample size for the first objective (prevalence of *CYP2D6* SNPs among Saudi SLE patients) was calculated using an online calculator based on the following assumptions: confidence level of 80%, error margin of 5%, population portion (18%) of *CYP2D6* ^*^ *41* among population size of approximately 2000 adult Saudis with SLE.^10,11^ Moreover, the sample size for the second objective (correlation between *CYP2D6* genotypes and SLE features) was calculated using G*Power software (version 3.1.9.7) based on the following parameters: 2-tailed test, alpha error probability of 0.05, power (1 minus beta error probability) of 0.80, and correlation effect for the alternative hypothesis of 0.30 (medium-sized effect size was used based on conventional Cohen’s standard for Chi-square contingency test).

Research ethical approval for this study was acquired from the Institutional Review Board of King Saud University in Riyadh, Saudi Arabia (approval no.: E-19-3955). Written informed consent was obtained from all participants according to the IRB of KSU guidelines. All study procedures were in accordance with the principles of Helsinki Declaration.

Participants’ characteristics were collected from the electronic health records database according to a standardized clinic procedure at KKUH, Riyadh, Saudi Arabia, and the SLE cohort protocol described previously.^12^ The collected data included age, gender, and clinical results for SLE.

A 4-mL sample of peripheral blood from each patient was collected into ethylenediaminetetraacetic acid-treated tubes after an overnight fast. The blood samples were immediately centrifuged at 2,000xg for 20 minutes at 10-15^o^C to obtain a buffy coat sample, then aliquoted appropriately and stored at -80^o^C until further testing.

The total genomic deoxyribonucleic acid (DNA) was extracted from 200 μL of buffy coat samples using the QIAamp Genomic DNA Blood kit (QIAGEN, Minneapolis, MN, USA) according to the manufacturer’s protocol. The eluted genomic DNA samples were then diluted to 20 ng/μL prior to the genotyping process. The quantity and quality of the extracted genomic DNA was evaluated using a NanoDrop™ 2000 spectrophotometer (Thermo Fisher Scientific Inc., Waltham, MA, USA).

The TaqMan™ SNP Genotyping kits including TaqMan^®^ SNP Genotyping Master Mix and Assays were purchased from Applied Biosystems (Thermo Fisher Scientific Inc., Waltham, MA, USA). The assays included: C__32407252_30 for rs1080985, C__27102448_30 for rs28624811, C__11484460_40 for rs1065852, C__34816116_20 for rs28371725, and C__27102414_10 for rs1135840.

The working stock per reaction well was prepared at a final volume of 20.00 μL, including 12.00 μL of TaqMan^®^ master mix, 0.60 μL of TaqMan^®^ assay, 6.40 μL of nuclear-free water, and 2.00 μL of gDNA sample. For assay C__27102448_30, the working stock per reaction well was prepared at a final volume of 10.00 μL, including 3.00 μL of TaqMan^®^ master mix, 5.75 μL of TaqMan^®^ assay, 0.25 μL of nuclear-free water, and 1.00 μL of DNA sample.

The polymerase chain reaction (PCR) condition was set for 40 cycles as follows: polymerase activation for 10 minutes at 95^o^C; dsDNA denaturation for 15 seconds at 95^o^C; and annealing/extension for 90 seconds at 60^o^C. For assay C__27102448_30, the PCR condition was set for 50 cycles as follows: polymerase activation for 10 minutes at 95^o^C; dsDNA denaturation for 15 seconds at 95^o^C; and annealing/extension for one minute at 60^o^C. The PCR and fluorescence measurements were carried out using a ViiA 7 Real-Time PCR system (Thermo Fisher Scientific Inc., Waltham, MA, USA) according to the manufacturer’s instructions.

For genotyping error rate checking, 30 samples (5% for each SNP) were chosen randomly to be re-genotyped blindly following the above method. The allelic differences between genotypes obtained were evaluated for any inconsistencies.

### Statistical analysis

The collected dataset included randomly missing values. Thus, multiple imputations using the Mersenne Twister method were applied. Descriptive analysis was used to describe participants’ general and clinical characteristics. Continuous variables were expressed as mean ± standard deviation (SD), while non-continuous (nominal) variables were expressed as proportion and percentage. The Fisher’s Exact test with mid-*P* values adjustment was carried out to evaluate the *CYP2D6* genotypes deviation from the Hardy-Weinberg equilibrium. Moreover, the crosstabulation method using a Chi-square test of independence (2x2 contingency test) was utilized to examine the relationship between the *CYP2D6* genotypes and SLE features. The Bonferroni correction method of Z-test was used to adjust the computed *P*-values. Furthermore, multiple univariate logistical regression models were employed to analyze the association of the variant *CYP2D6* genotypes as predictors (independent variables) for SLE features (dependent variables). For the adjusted models, age and gender were considered covariates. A bootstrap test based on 1000 samples (bias-corrected and accelerated method) was carried out to verify statistically significant findings. All statistical analyses were evaluated with 95% confidence intervals (CI). A *p*-value of <0.05 was considered significant. The statistical analyses were carried out using the Statistical Package for the Social Sciences, version 27.0 (IBM Corp., Armonk, NY, USA).

## Results

A total of 107 participants with confirmed SLE were included in this study. Table 2 shows the distribution of the selected *CYP2D6* SNPs in our study sample. All of the selected *CYP2D6* SNPs were consistent with Hardy-Weinberg equilibrium, except for rs1065852 (*p*<0.001). Nevertheless, the genotypes and alleles showed significant genotypic variability, where the frequencies of the least common variants were more than 1%.

**Table 2:** - Genotype and allele frequencies of the selected *cytochrome P450 2D6* single nucleotide polymorphisms in the study sample (N=107).

SNPs	Genotypes (N=107)			Alleles (N=214)		*P*-values (for HWE)
rs1080985	G/G	G/C	C/C	G	C	0.90
	56 (52.3)	43 (40.2)	8 (7.5)	155 (72.4)	59 (27.6)	
rs28624811	G/G	G/A	A/A	G	A	0.77
	30 (28.0)	52 (48.6)	25 (23.4)	112 (52.3)	102 (47.7)	
rs1065852	G/G	G/A	A/A	G	A	<0.001
	80 (74.8)	14 (13.1)	13 (12.2)	174 (81.3)	40 (18.7)	
rs28371725	C/C	C/T	T/T	C	T	0.86
	76 (71.0)	29 (27.1)	2 (1.9)	181 (84.6)	33 (15.4)	
rs1135840	G/G	G/C	C/C	G	C	0.36
	40 (37.4)	55 (51.4)	12 (11.2)	135 (63.1)	79 (36.9)	
Values are presented as numbers and precentages (%). *P*-values above 0.05 represents consistency of *CYP2D6* genotypes with HWE principle. HWE: Hardy-Weinberg equilibrium, SNPs: single nucleotide polymorphisms						

In our study sample, for rs1080985 (*CYP2D6* ^*^ *2A*), the G allele was identified as dominant and the C allele as a variant. For rs28624811 (*CYP2D6* ^*^ *36*), the G allele was identified as dominant and the A allele as a variant. For rs1065852 (*CYP2D6* ^*^ *4*), the G allele was identified as dominant and the A allele as a variant. For rs28371725 (*CYP2D6* ^*^ *41*), the C allele was identified as dominant and the T allele as a variant. For rs1135840 (*CYP2D6* ^*^ *10*), the G allele was identified as dominant and the C allele as a variant.

Overall, the most predominant variant genotype (AA) was in rs28624811 (23.4%) within the promoter region, and the least prevalent variant genotype (TT) was in rs28371725 (1.9%) within the intronic region of *CYP2D6* gene (Table 2).

Table 3 shows the observed distribution of the patient characteristics per *CYP2D6* SNP genotypes. Among our study sample, most participants were females (85%) in their reproductive age. Overall, the mean age for our study sample was 37.59±11.3 years. Considering their clinical characteristics, the most common manifestations were immunological disorders (86%), followed by arthritis (80.4%), and cutaneous involvements (77.6%). Furthermore, Table 3 shows the results from the Chi-square test used to analyze the relationship between the *CYP2D6* genotypes and SLE features. Results indicated a statistically significant correlation between rs1080985 SNP and serositis manifestation, as well as between rs28624811 SNP and renal involvement (*p*=0.03).

**Table 3:** - Characteristics of the study sample stratified by the *cytochrome P450 2D6* single nucleotide polymorphism genotypes.

Variables	*CYP2D6* genotypes									
	rs1080985		rs28624811		rs1065852		rs28371725		rs1135840	
	0 (n=56)	1 (n=51)	0 (n=30)	1 (n=77)	0 (n=80)	1 (n=27)	0 (n=76)	1 (n=31)	0 (n=40)	1 (n=67)
***Age, mean±SD***	38.5±10.4	36.6±12.2	37.1±10.1	37.8±11.8	38.7±11.4	34.3±10.8	37.6±11.1	37.5±12.1	35.4±11.1	38.9±11.3
*P*-values	0.37		0.77		0.08		0.97		0.12	
***Gender (n=91)***	48	43	26	65	67	24	67	24	34	57
*P*-values	0.84		0.77		0.52		0.16		0.99	
***Cutaneous (n=83)***	45	38	25	58	61	22	59	24	31	52
*P*-values	0.47		0.37		0.57		0.98		0.99	
***Arthritis (n=86)***	44	42	23	63	62	24	61	25	33	53
*P*-values	0.62		0.55		0.20		0.96		0.67	
***Serositis (n=20)***	6	14	4	16	18	2	15	5	8	12
*P*-values	0.03		0.37		0.08		0.66		0.79	
***Renal (n=50)***	29	21	19	31	35	15	38	12	20	30
*P*-values	0.27		0.03		0.29		0.29		0.60	
***Neurological (n=19)***	7	12	2	17	16	3	13	6	10	9
*P*-values	0.14		0.06		0.30		0.78		0.13	
***Hematological (n=47)***	23	24	14	33	32	15	34	13	22	25
*P*-values	0.53		0.72		0.16		0.79		0.07	
***Immunological (n=92)***	45	47	24	68	68	24	66	26	36	56
*P*-values	0.08		0.27		0.61		0.69		0.35	
Values are presented as numbers or proportion in a subgroup. *P*-values (Bonferroni-adjusted) below 0.05 represents a statistically significant relationship (contingency) between *CYP2D6* genotypes and participant characteristics. *CYP2D6*: *cytochrome P450 2D6*, SD: standard deviation, SNP: single nucleotide polymorphism, *CYP2D6* SNP genotype 0: reference variant (homozygous dominant), *CYP2D6* SNP genotype 1: alternative variants (heterozygous and homozygous recessive)										

Table 4 shows the results of logistical regression analysis for the association of *CYP2D6* polymorphisms as explanatory variables for the different SLE features. We found that the rs1080985 genotypes were significantly associated with the presence of serositis (*p*=0.03). The odds of having serositis were 3.15 times higher in those carrying the variant genotype than in those carrying the dominant genotype. Also, we found that the rs28624811 genotypes were associated significantly with renal involvement (*p*=0.03). The odds of having renal involvement were 0.39 times higher in those carrying the variant genotype compared to those carrying the dominant genotype, whereas the odds of having renal involvement were much higher in those carrying the dominant genotype compared to those carrying the variant genotype (OR=2.56; 95% CI=[1.07-6.13]; *p*=0.03). These associations remained statistically significant even after adjusting for age and gender (*p*<0.05).

**Table 4:** - Univariate logistical regression of systemic lupus erythematosus feature (dependent variable) on recessive *cytochrome P450 2D6* genotype (explanatory variable).

Variables	Unadjusted model			Adjusted model*		
	OR	95% CI	*P*-values	OR	95% CI	*P*-values
***Cutaneous***						
rs1080985 (1)	0.71	0.29-1.78	0.47	0.65	0.25-1.68	0.38
rs28624811 (1)	0.61	0.21-1.82	0.38	0.63	0.21-1.95	0.43
rs1065852 (1)	1.37	0.46-4.11	0.57	1.08	0.34-3.40	0.90
rs28371725 (1)	0.99	0.36-2.69	0.98	1.08	0.38-3.08	0.89
rs1135840 (1)	1.01	0.39-2.57	0.99	1.20	0.45-3.20	0.72
***Arthritis***						
rs1080985 (1)	1.27	0.49-3.33	0.62	1.29	0.47-3.51	0.62
rs28624811 (1)	1.37	0.49-3.82	0.55	1.48	0.51-4.31	0.47
rs1065852 (1)	2.32	0.63-8.61	0.21	2.09	0.54-8.11	0.28
rs28371725 (1)	1.02	0.36-2.94	0.96	1.27	0.42-3.91	0.67
rs1135840 (1)	0.80	0.29-2.20	0.67	0.83	0.29-2.36	0.72
***Serositis***						
rs1080985 (1)	3.15	1.11-8.98	0.03	3.01	1.05-8.66	0.04
rs28624811 (1)	1.70	0.52-5.59	0.38	1.71	0.52-5.66	0.38
rs1065852 (1)	0.28	0.06-1.28	0.10	0.24	0.05-1.13	0.07
rs28371725 (1)	0.78	0.26-2.38	0.66	0.73	0.23-2.27	0.58
rs1135840 (1)	0.87	0.32-2.36	0.79	0.95	0.35-2.63	0.93
***Renal***						
rs1080985 (1)	0.65	0.3-1.40	0.27	0.59	0.27-1.30	0.19
rs28624811 (1)	0.39	0.16-0.93	0.03	0.39	0.16-0.94	0.04
rs1065852 (1)	1.61	0.67-3.87	0.29	1.39	0.56-3.43	0.48
rs28371725 (1)	0.63	0.27-1.48	0.29	0.62	0.26-1.50	0.29
rs1135840 (1)	0.81	0.37-1.78	0.60	0.92	0.41-2.06	0.83
***Neurological***						
rs1080985 (1)	2.15	0.77-5.99	0.14	2.11	0.75–5.91	0.16
rs28624811 (1))	3.97	0.86-18.36	0.08	4.13	0.89–19.24	0.07
rs1065852 (1	0.50	0.13-1.87	0.30	0.43	0.11–1.66	0.22
rs28371725 (1)	1.16	0.40-3.40	0.78	1.22	0.41–3.63	0.72
rs1135840 (1)	0.47	0.17-1.27	0.13	0.49	0.18–1.35	0.17
***Hematological***						
rs1080985 (1)	1.28	0.59-2.74	0.53	1.18	0.54-2.60	0.67
rs28624811 (1)	0.86	0.37-2.00	0.72	0.87	0.37-2.07	0.75
rs1065852 (1)	1.87	0.78-4.53	0.16	1.63	0.66-4.03	0.29
rs28371725 (1)	0.89	0.38-2.08	0.79	0.88	0.37-2.12	0.78
rs1135840 (1)	0.49	0.22-1.08	0.08	0.54	0.24-1.22	0.14
***Immunological***						
rs1080985 (1)	2.87	0.85-9.68	0.09	3.00	0.87-10.31	0.08
rs28624811 (1)	1.89	0.61-5.86	0.27	1.96	0.62-6.19	0.25
rs1065852 (1)	1.41	0.37-5.44	0.62	1.34	0.34-5.30	0.68
rs28371725 (1)	0.79	0.25-2.53	0.69	0.88	0.27-2.89	0.83
rs1135840 (1)	0.57	0.17-1.91	0.36	0.56	0.16-1.92	0.36

* Model adjusted for age and gender. P-values below 0.05 represents a statistically significant association between clinical features of systemic lupus erythematosus and variant *CYP2D6* genotypes. Bootstrap test based on 1000 samples (bias corrected and accelerated method) was carried out to verify statistically significant findings. CI: confidence interval, OR: odds ratio, SNP: single nucleotide polymorphism, *CYP2D6* SNP genotype (1): heterozygous or homozygous recessive versus homozygous dominant genotypes

It is worth mentioning that there were statistically trending risk associations between the *CYP2D6* SNPs in the promoter region and SLE clinical features. For instance, we found that carrying the variant rs28624811 genotype could possibly be associated with neurological manifestation (OR=3.97; 95% CI=[0.86-18.36]; *p*=0.08). Also, we found that carrying the variant rs1080985 genotype could possibly be associated with immunological manifestation (OR=2.87; 95% CI=[0.85-9.68]; *p*=0.09). However, these associations remained insignificant even after adjusting for age and gender (*p*>0.05).

## Discussion

The present study provides a new perspective on lupus-associated genetic loci. Herein, we selected 5 SNPs in different loci on the *CYP2D6* gene. Through genotyping, we identified the prevalence of the selected SNPs among our study sample. Overall, we found that *CYP2D6* SNP rs28624811 (*CYP2D6* ^*^ *36*) within the promoter gene region was prevalent in Saudis with SLE. Also, we found a possible correlation between the *CYP2D6* polymorphisms and SLE manifestations. Perhaps the generation of deficient CYP enzymes leading to disturbed endogenous and exogenous metabolism might be responsible for determining the trend of manifestations in SLE.^13^

The overall *CYP2D6* allelic frequencies in our study were incongruent with the general population reported previously.^2,3^ For instance, throughout the Middle Eastern populations, *CYP2D6* ^*^ *2* (22%) and *CYP2D6* ^*^ *41* (20%) were the most common alleles, while *CYP2D6* ^*^ *4* (8%) and *CYP2D6* ^*^ *10* (6%) were the least prevalent alleles. We found that *CYP2D6* ^*^ *10* (37%) and *CYP2D6* ^*^ *2* (28%) were more common alleles, while *CYP2D6* ^*^ *4* (19%) and *CYP2D6* ^*^ *41* (15%) were less prevalent alleles. Our findings indicated that functionally significant variants are prevalent among Saudi SLE patients, suggesting that most of them are predicted to have inactive or decreased activity. A similar metabolic deviation was also evident in other ethnic groups, such as Jewish populations.^2^ This is presumably due to the high consanguinity (high inbreeding coefficient) leading to interethnic haplotype variability.^14^ However, a fair representation of these populations remains insufficient to draw such presumptions.

Among all the Middle Eastern populations, for instance, the highest prevalence (32%) of *CYP2D6* ^*^ *2* was among Iranian and Eastern Azerbaijan populations.^15^ Similarly, high frequencies were reported in Turkey and Syria.^16,17^ Meanwhile, the lowest prevalence (9%) was reported among nomadic groups in the Israel/Palestine region.^18^ Likewise, low frequencies (<16%) were reported in Saudi Arabia, United Arab Emirates, and Ethiopia.^19-21^ Our results of *CYP2D6* ^*^ *2* for SLE patients were higher than other local studies.^19,20^ Furthermore, we demonstrated the prevalence of a new SNP (rs28624811) located in the promoter region of the *CYP2D6* gene. Interestingly, results showed that the variant allele *CYP2D6* ^*^ *36* (48%) was frequently found among our study sample compared to the other selected *CYP2D6* SNPs. We speculate that the occurrence of this variant within the core regulatory region of the *CYP2D6* promoter might affect the transcription factor binding, which alters the promoter activity in gene transcription, mRNA stability, and translation. Subsequently, this may alter the enzyme level responsible for endogenous and exogenous metabolism, potentially contributing to different clinical outcomes.

Furthermore, our results from the regression analysis showed a statistically significant association between *CYP2D6* ^*^ *2* and serositis. The *CYP2D6* has been identified in smooth tissue membranes.^22,23^ However, its interaction with the bilayer lipid membrane is yet to be explored. We speculate that *CYP2D6* might exhibit similar functional characteristics to *cytochrome P450 2J2* (*CYP2J2*) found in cardiovascular tissues. The *CYP2J2* is involved in the metabolism of arachidonic acid to epoxyeicosatrienoic acids, which are anti-migratory, anti-proliferative, and anti-inflammatory responses in endothelial cells.^24^ Thus, genetic polymorphism may have functional consequences leading to an increased risk of heart disease. In fact, some studies on the Saudi population have reported that hypertensive patients showed a significantly higher frequency of *CYP2J2* ^*^ *7* and *CYP2D6* ^*^ *10* compared to patients with normal blood pressure.^25^

Additionally, we found a statistically significant association between *CYP2D6* ^*^ *36* and renal manifestations. In a case study, Leung et al^26^ reported on 2 patients with acute renal damage that suffered multiple drug allergies/intolerances.^27^ In both cases, the renal injury was believed to be related to increased drug exposure leading to nephrotoxicity due to poor metabolizer of CYP polymorphism. Other studies also demonstrated that polymorphic CYPs might promote renal cancer development by downregulating the death-associated protein kinase-1.^28,29^ Thus, further investigation of SLE needs to account for CYP polymorphisms for individualized medicine practices.^30^ Taken together, the genetic polymorphisms within the promoter region of the *CYP2D6* gene could be potential predictors for certain SLE features. However, further research on a large population must confirm these results.

The trends for *CYP2D6* ^*^ *4* frequencies in the Middle East pivot between 3-12%, as confirmed by meta-analysis studies.^2,31^ Our observed frequency of *CYP2D6* ^*^ *4* among our study sample was above the previously reported data. However, recent data from regional studies did not support our findings. For instance, approximately 28% (on average) of Egyptians were reported to carry the recessive allele of *CYP2D6* ^*^ *4*.^32-34^ Moreover, Alkreathy et al^19^ found that 100% of their Saudis subjects had the *CYP2D6* ^*^ *4* allele. The authors of these studies did not explain their divergent results, though methodology differences may be important to consider.

Our advent finding that the genotypes at rs1065852 deviated from the Hardy-Weinberg equilibrium also should not be neglected. Although a re-genotyping experiment was carried out to confirm our results, there were zero missing calls or allelic differences between the repeated samples. In fact, the overall genotyping quality for SNP rs1065852 was 98.2%. Additionally, population stratification may explain the deviation. We found that genotyping results for individuals with presumably African ancestral backgrounds drove the genotypic distribution towards equilibrium (*p*=0.05) but not for the remaining individuals. This finding may support the existence of unique genetic profiles for individuals with Arab ancestral backgrounds. However, other unknown confounding factors might have remained unaccounted for within the scope of our study.

Another interesting finding was the identification of *CYP2D6* ^*^ *41*. In a recent meta-analysis review, the *CYP2D6* ^*^ *41* allele was relatively more common throughout the Middle East compared to other regions worldwide.^3^ The highest percentages were seen among Palestinians (29%), followed by Saudi Arabians (18%).^10,18^ Our findings of *CYP2D6* ^*^ *41* contradicted these studies, which was the least frequent variant in our study sample. This may support the existence of a unique genetic profile of Saudi SLE, especially considering the homogeneity of our sample population (namely, subjects with similar diagnoses and ethnic backgrounds). However, further research on a large population is needed to confirm these results.

The overall percentage of *CYP2D6* ^*^ *10* found among our study sample was higher than the pooled data from Middle Eastern populations.^2,3^ However, we found that the pattern of the C allele to the G allele among our study sample was similar to that of the global data reported in the Allele Frequency Aggregator project database at the National Center for Biotechnology Information (release version: 20201027095038). Thus, it should not be excluded that other mutations (such as *CYP2D6* ^*^ *4* or *CYP2D6* ^*^ *41*) could be in linkage disequilibrium with *CYP2D6* ^*^ *10* and play a role in the phenotypic effect.^35^

Intriguingly, we detected that SLE patients carrying the dominant rs1135840 genotype (GG) had slightly higher neurological (OR=2.15; 95% CI=[0.79-5.86]; *p*=0.14) and hematological (OR=2.05; 95% CI=[0.93-4.55]; *p*=0.08) involvement compared to those carrying the variant genotype (Table 4). The role of *CYP2D6* in the brain varies from the metabolism of endogenous compounds, such as dopamine and serotonin, to interactions with numerous drugs that are of potential clinical importance for neurological and cognitive disorders.^36,37^ Moreover, recent studies showed the association of *CYP2D6* ^*^ *10* with an increased risk of hepatological and hematological toxicity as a result of drug-related adverse reactions.^38^ Interestingly, a recent study investigating the *CYP2D6* ^*^ *10* in SLE patients showed that the GG genotype had decreased activity.^39^ Similarly, Lee et al^40^ found that carriers of the GG genotypes in rs1134840 had elevated levels of norclozapine. We speculate that the alleles of importance may vary between populations (namely, population-dependent), which needs to be considered in clinical research or patient care.

### Study limitations

First, not all factors have been taken into account systematically. Nevertheless, genetic variants are far less susceptive to confounding bias than most non-genetic measures. Thus, our results merely provide support for a possible link between *CYP2D6* genotypes and SLE criteria. Second, the Saudi population has unique variations compared with other Arab ethnicities in the region. Our subject recruitment method from the National Lupus Cohort offered a fair representation of the population. Though, the racial admixture and high consanguinity of the Saudi population should not be neglected in genetic studies. Third, the recruitment of our study sample was restricted by the COVID-19 pandemic; thus, the small sample size might have limited power. To circumvent the potential bias stemming from false discovery, we carried out an a priori power inquiry to determine a reasonable sample size, then carried out a post-hoc power analysis to confirm the conferred statistical significance. Nevertheless, the results of this investigation should be interpreted with caution. We intend to carry out the experiment using the whole-gene sequencing experiment on a larger longitudinal SLE cohort to confirm the study findings.^12^

In conclusion, we identified a common *CYP2D6* polymorphism in Saudi SLE patients. This finding may support the existence of unique genetic profiles for SLE patients with Saudi ancestral backgrounds. Also, we found several *CYP2D6* SNPs possibly related to the different clinical features of SLE. This a priori knowledge might be a useful reference in potentially determining genetic factors of SLE-related dysfunctions and may help understand the likely pathophysiological link between the SLE manifestations. Future genetics studies on this topic may lead to alternative disease intervention strategies for these patients.
